# Supplementary material for: The clinical features and operation experience of multilobar involved congenital lung malformation: A retrospective cohort study
Source: PLoS One. 2024 Oct 24;19(10):e0312592. doi: 10.1371/journal.pone.0312592 (PMC11500879; doi:10.1371/journal.pone.0312592)
Supplement: S1 File — (PDF) [file pone.0312592.s001.pdf]

Multilobar group:

ID	gender	age(month)	BMI	classification	Prenatal diagnosis	preoperation complication	blood loss(ml)	operation time(min)	post-los(day)	chest tube(day)	postoperation complication
0014XXXXX3	0	5	15.22	CPAM	1	infection	10	85	6	2	
0018XXXXX7	0	12	15.29	CPAM	1	infection	15	107	7	1	
0018XXXXX9	1	6	16.80	CPAM	1		7	79	3	1	
0018XXXXX4	1	100	15.36	CPAM	0	pneumothorax	10	130	4	1	
0018XXXXX5	0	9	17.30	CPAM	1		15	95	3	2	
0019XXXXX7	0	40	16.86	CPAM	0		20	98	4	3	
0019XXXXX1	1	8	17.31	CPAM	1		15	68	4	4	Pneumonia
0019XXXXX3	1	9	17.36	CPAM	1		8	78	4	3	
0007XXXXX5	1	6	15.71	CPAM+PS	1	infection	8	76	11	7	Atelectasis
0009XXXXX2	1	5	15.88	CPAM	1		15	107	6	1	
0010XXXXX6	1	11	17.36	CPAM	1	infection	15	178	12	2	
0016XXXXX5	0	9	18.37	CPAM	1		10	78	5	2	
0018XXXXX4	1	7	15.22	CPAM	1		10	83	4	1	
0019XXXXX9	1	9	15.95	CPAM	1		15	99	5	1	
0019XXXXX2	1	6	17.35	CPAM	1		15	90	4	1	
0019XXXXX6	1	13	18.75	CPAM	0		10	96	8	2	
0022XXXXX1	1	7	15.22	CPAM+PS	1		15	78	3	1	
0015XXXXX6	0	12	13.85	CPAM	1	pneumothorax	25	113	9	8	
0015XXXXX6	1	17	15.84	CLE	1		10	87	4	2	
0019XXXXX0	1	6	17.22	CPAM	1		8	80	5	2	
0015XXXXX6	1	12	14.37	CLE	1		10	88	4	2	
0020XXXXX2	1	10	17.83	CPAM	1	infection	30	150	14	5	Pneumonia
0020XXXXX4	0	44	16.02	CPAM	0		8	78	4	1	
0013XXXXX7	0	11	15.50	CPAM	1	hydrothorax	20	86	7	2	
0017XXXXX5	0	7	16.22	CPAM+PS	1		15	91	5	1	
0017XXXXX6	1	6	17.35	CPAM	1	infection	8	105	9	2	
0017XXXXX7	1	18	16.09	CPAM	0		8	78	6	2	
0018XXXXX6	1	8	14.29	CPAM	1		15	95	4	1	
0015XXXXX5	1	28	13.72	CLE	0	hemoptysis	10	108	3	1	
0019XXXXX2	0	7	15.59	CLE	1		18	105	4	1	
0019XXXXX2	1	8	15.22	CPAM	1		10	79	3	1	
0015XXXXX6	1	30	14.58	CLE	0	infection	20	86	21	7	Air leakage
0017XXXXX4	1	143	16.61	CPAM	0	infection	15	96	7	4	
0033XXXXX4	1	11	15.98	CPAM	1		10	102	7	3	
	0:Female 10		Mean:				Mean:	Mean:			
	1: male 24	Median:	16.11				13.32352941	95.64705882	Median:	Median:	
		11	Standard Deviation:		26(76.4%)	12(35.2%)	Standard Deviation:	Standard Deviation:	5	2	4(11.7%)
			1.23				5.205882353	21.70237498			

Unilobar group:

ID	gender	age(month)	BMI	classification	Prenatal diagnosis	preoperation complication	blood loss(ml)	operation time(min)	post-los(day)	chest tube(day)	postoperation complication
0021XXXXX4	0	4	12.65	CPAM	1		5	40	3	0	
0021XXXXX3	0	7	11.22	CPAM	1		4	53	3	0	
0032XXXXX9	1	17	15.88	PS	1		5	47	3	0	
0009XXXXX9	1	5	13.45	CPAM	1		5	45	4	1	
0032XXXXX9	0	6	13.02	CPAM	1		10	60	4	1	
0032XXXXX2	1	21	12.85	CPAM	1	infection	12	50	4	3	
0032XXXXX7	1	10	12.29	CPAM	1		5	56	3	1	
0018XXXXX4	0	5	12.72	CPAM	1		5	39	4	1	
0032XXXXX1	1	3	15.22	CPAM	1		5	49	5	1	
0032XXXXX3	1	6	15.95	CPAM	1		8	45	3	0	
0021XXXXX5	1	7	14.35	CPAM	1		10	39	3	0	
0032XXXXX2	0	10	15.75	PS	1		10	50	3	0	
0032XXXXX4	0	18	11.35	CPAM	1		5	40	3	0	
0032XXXXX2	1	78	15.22	CPAM	0		5	39	3	0	
0032XXXXX3	1	35	13.50	CPAM	0		7	48	4	1	
0032XXXXX8	1	5	12.22	CPAM	1		5	47	4	1	
0032XXXXX0	0	6	12.35	CPAM	1	infection	15	70	6	5	
0032XXXXX9	0	12	14.09	CPAM	1		6	44	5	1	
0032XXXXX8	0	7	14.29	CPAM	1		5	42	3	0	
0032XXXXX0	1	9	13.72	PS	1		6	40	3	0	
0032XXXXX9	1	16	14.09	CPAM	1		9	43	3	0	
0032XXXXX9	0	6	15.02	PS	1		10	50	4	1	
0032XXXXX9	0	19	13.58	CPAM	0	infection	26	92	8	4	Air leakage
0021XXXXX0	1	5	16.01	CPAM	1		6	42	5	1	
0032XXXXX8	0	6	12.29	CLE	1		5	45	5	2	
0032XXXXX0	1	20	12.96	PS	1		6	41	4	1	
0032XXXXX4	1	11	12.30	CPAM	1		7	45	3	0	
0032XXXXX6	0	6	12.86	CPAM	1		5	29	2	0	
0019XXXXX5	0	7	13.29	CPAM	1		8	56	3	0	
0032XXXXX4	0	18	13.90	CPAM	1		5	44	4	1	
0020XXXXX7	1	43	12.36	CPAM	0	infection	15	52	3	0	
0032XXXXX2	0	6	14.08	CPAM	1		6	47	4	1	
0021XXXXX1	1	7	13.09	CPAM	1		5	50	3	0	
0032XXXXX3	0	13	13.72	CPAM	0		6	40	3	2	
0032XXXXX0	1	4	11.85	CPAM	1		7	45	3	0	
		Median:	Mean:				Mean:	Mean:	Median:	Median:	
	0:Female 15	7	13.53		29(85.2%)	4(11.7%)	7.542857143	47.54285714	3	1	1(2.9%)
	1: male 19		Standard Deviation:				Standard Deviation:	Standard Deviation:			
			1.286827515				4.197569754	10.45901623			
